# FFNT25 ameliorates unilateral ureteral obstruction-induced renal fibrosis

**DOI:** 10.1080/0886022X.2019.1612430

**Published:** 2019-05-29

**Authors:** Wen Li, Yue Lu, Yan Lou, Shiyue Zhao, Wenpeng Cui, Yangwei Wang, Manyu Luo, Jing Sun, Lining Miao

**Affiliations:** Department of Nephropathy, The Second Hospital of Jilin University, Changchun, China

**Keywords:** FFNT25, UUO, renal fibrosis, α-SMA, PAI-1

## Abstract

Renal fibrosis is a common pathological feature of chronic kidney disease (CKD) patients who progress to end-stage renal disease (ESRD). With the increasing incidence of CKD, it is of importance to develop effective therapies that blunt development of renal fibrosis. FFNT25 is a newly developed molecular compound that could be used to prevent fibrosis. In this study, we administered FFNT25 to rats following unilateral ureteral obstruction (UUO) to investigate its anti-fibrosis mechanism. Thirty-two Sprague-Dawley rats were randomly divided into four groups: (1) control (normal rats), (2) sham-operated, (3) UUO-operated + vehicle, and (4) UUO-operated + FFNT25. Two weeks after UUO, the rats were gavaged with either FFNT25 (20.6 mg/kg/day) or vehicle for two weeks. Serum, urine, and kidney samples were collected at the end of the study. FFNT25 reduced levels of renal fibrosis and decreased mRNA and protein levels of extracellular matrix (ECM) markers α-smooth muscle actin (α-SMA) and plasminogen activator inhibitor-1 (PAI-1) following UUO compared to vehicle treatment (*n* = 8, *p*<.05). The current results indicate that FFNT25 can affect both the production and degradation of collagen fibers to reduce fibrosis.

## Introduction

Renal fibrosis is characterized by infiltration of inflammatory cells, deposition of extracellular matrix (ECM), and differentiation and activation of myofibroblasts in the kidney [1,2]. When renal fibrosis develops, most chronic kidney disease (CKD) patients will progress to end-stage renal disease (ESRD), in which kidney transplantation is the only therapeutic option. It has been reported that 10% of the world’s population suffer from CKD [3,4]. Thus, effective therapy that blunts development of renal fibrosis in CKD is of clinical importance.

Generally, renal matrix deposition is controlled by the balance of ECM production and degradation. In renal fibrosis, ECM is mainly produced by myofibroblasts [5–7], but the underlying mechanisms remain to be investigated [8–10]. However, increased levels of α-smooth muscle actin (α-SMA) have been observed following myofibroblast activation [6,11], which promotes ECM production. Plasminogen activator inhibitor-1 (PAI-1), a major inhibitor of plasminogen activation, regulates both fibrinolysis and activation of matrix metalloproteinases (MMPs) [12–14]. Under physiological conditions, PAI-1 is produced in little amounts, while in injured tissues its expression increased significantly to accelerates wound healing [15,16]. Persistent overexpression of PAI-1 reduces ECM degradation via perturbing the plasminogen activation system, which leads to multiple organ fibrosis [17]. Several studies indicated that inhibition of PAI-1 could ameliorate organ fibrosis [18,19]. Therefore, PAI-1 has been proposed as a potential target for controlling renal fibrosis since inhibiting PAI-1 can accelerate ECM degradation [20,21].

Numerous molecular pathways involved in renal fibrosis have been confirmed in preclinical studies. The small molecule compound, pirfenidone (PFD), has been shown to effectively manage renal fibrosis in animal models [22,23]. In a phase 2 clinical study, PFD significantly ameliorated the decline in glomerular filtration rate (GFR) in patients with focal segmental glomerulosclerosis [24]. However, PFD treatment failed to improve GFR in the placebo-controlled phase 1 and 2 clinical studies that included diabetic patients [25]. Thus, unfortunately, due to poor clinical translation, there are still no clinically approved drugs that specifically target renal fibrosis. In view of this, more efforts are needed to translate current basic scientific research into clinical applications and develop effective renal anti-fibrosis drugs.

Based on the anti-fibrosis effect of PFD and its limitation, Sunshine Lake Pharma Co. (Guangdong, China) structurally modified PFD and recently independently developed another small molecule named FFNT25 (see detailed properties in Table 1). In a trial conducted by Sunshine Lake Pharma Co. (Dongguan, China), both FFNT25 (1.7 mM) and PFD (5.8 mM) effectively inhibited the activation of renal fibroblasts in cultured renal fibroblast BHK-21 cells. In this study, we further investigated the anti-fibrotic potential of FFNT25 *in vivo*. Renal fibrosis was induced in Sprague-Dawley (SD) rats by unilateral ureteral obstruction (UUO) [26]. FFNT25 was administered two weeks after UUO when the rats exhibited significant renal fibrosis. The current results clearly indicate that FFNT25 is a potential therapeutic option for controlling renal fibrosis.

**Table 1.** Molecular structure and physical properties of FFNT25.

Item	FFNT25	Structure
Name	1-(3-Fluoro benzyl)-5-methyl pyrimidine-2(1H)-ketone	
Molecular formula	C_12_H_11_FN_2_O	
Molecular weight	218.23	
Appearance	White solid	
Stereo structures	No chiral center, without optical activity	

## Materials and methods

### Experimental animals and protocols

All animal protocols were approved by the Animal Care and Use Committee at the Second Hospital of Jilin University (Jilin, China, Permit No. 2018178). Male SD rats (8 weeks old, weighing 200 ± 20 g; *n* = 32) were purchased from the Animal Center at Jilin University. The rats were housed in a temperature-controlled room (21 ± 2 °C) at the Animal Center with a 12-h light:12-h dark cycle. After a one-week acclimatization period, the rats were randomly and equally divided into four groups: (1) control (normal rats), (2) sham-operated, (3) UUO-operated + vehicle, and (4) UUO-operated + FFNT25. UUO was performed under complete chloral hydrate anesthesia (200 mg/kg, intraperitoneally) by ligating the left lateral ureter. Two weeks after UUO, the rats were gavaged with either FFNT25 (20.6 mg/kg/day) in vehicle or vehicle only for two weeks. Vehicle contained 3% kolliphor@HS15 (Sigma-Aldrich, St. Louis, MO) and 3% glycerol (Beijing Chemical Works, Beijing, China) dissolved in physiological saline. The chemical property of FFNT25 (Sunshine Lake Pharma Co., Guangdong, China) is shown in Table 1.

### Blood and urine assays

Plasma and urine levels of creatinine (Cr) and N-acetyl-β-d-glucosaminidase (NAG) were measured using commercially available colorimetric assay kits purchased from BioAssay Systems (Hayward, CA) and Lengton Bioscience Co. (Shanghai, China), respectively.

### Renal histopathological and immunohistochemical staining

Kidney samples were collected, fixed (10% phosphate-buffered formalin), and paraffin sectioned at 0.5-μm, 2.0-μm, and 3.0-μm for periodic acid-Schiff (PAS), Masson’s trichrome, and immunohistochemical staining, respectively. Tubulointerstitial injury, characterized by renal tubular dilation, tubular brush border loss, and tubular epithelial cell necrosis/loss, was scored as follows: 0, none; 0.5, <10%; 1, 10–25%; 2, 25–50%; 3, 50–75%, and 4, >75% [27]. Interstitial collagen deposition was calculated as the percentage of blue collagen area per total Masson’s trichrome staining [28]. The presence of collagen-I, collagen-III, collagen-IV, α-SMA, and PAI-1 was analyzed using immunohistochemical staining. All slides were observed by experienced pathological experts who were blinded to the study groups and were semi-quantified in at least 10 randomly chosen non-overlapping fields (×400) using Image J software (Version 6, The Java^TM^ Platform).

### Real-time quantitative PCR (qPCR)

Total RNA was extracted from renal tissues using the TRIZOL reagent (Invitrogen, Carlsbad, CA). The concentration and purity of the extracted RNA were determined using a NanoDrop ND-2000 spectrophotometer (Thermo Scientific, Waltham, MA). A total of 1 mg RNA was used for cDNA synthesis with a high-capacity cDNA reverse transcription kit according to the manufacturer’s protocol (Takara, Shenyang, China). Real-time qPCR was carried out using a SYBR^®^ Select Master Mix kit (Applied Biosystems^®^, Foster City, CA) and performed in triplicate in a final volume of 20 μL for each sample in the ABI 7300 Real-Time PCR system. Relative mRNA expression was normalized to β-actin using the 2^−ΔΔCt^ method. Primer sequences are listed in Table 2.

**Table 2.** Sequences of primers used in the study.

Genes	Forward (5′–3′)	Reverse (5′–3′)
Collagen-I	ACGTCCTGGTGAAGTTGGTC	TCCAGCAATACCCTGAGGTC
Collagen-III	GATCAGATGGTCAGCCAGGT	AGATGGACCAACAGGACCAG
Collagen-IV	GGGCTTTCCTGGTGAATCCG	ACCGGGCTCTCCTCTTAACC
Fibronectin	GATCTGCGATTCACCAATATCG	CTCGTTCTTCACAGGTGAGTAG
α-SMA	CTGAGCGTGGCTATTCCTTC	AGAAGAGGAAGCAGCAGTGG
PAI-1	GTATCGTCCTCCATTGCTATGA	AATGAGAAAAGTTTGTGGGTCG
β-Actin	ATGGTGGGTATGGGTCAGAA	TCCATATCGTCCCAGTTGGT

α-SMA: α-smooth muscle actin; PAI-1: plasminogen activator inhibitor-1.

### Western blot analysis

Western blot analysis was conducted using a method modified from a previous report [29]. Briefly, kidney tissues were homogenized in RIPA buffer (Beyotime Biotechnology Corp., Nanjing, China). Part of the supernatant was collected to determine protein concentration (Beyotime Biotechnology Corp., Nanjing, China), and the remaining supernatant was subjected to western blot analysis. Total protein was electrophoresed on 8% SDS-polyacrylamide gels (SDS-PAGE) and transferred onto a polyvinylidenedifluoride membrane. After being blocked with 5% nonfat dried milk, the membranes were incubated (4 °C, overnight) with anti-GAPDH (1:800 dilution, AF0006), anti-collagen-I (1:1000 dilution, NB600-408), anti-collagen-III (1:1000 dilution, ab7778), anti-collagen-IV (1:2000 dilution, ab6586), anti-fibronectin (1:2000 dilution, ab6328), anti-α-SMA (1:500 dilution, ab5694), and anti-PAI-1 (1:1000 dilution, ab66705) antibodies. The secondary antibody (horseradish peroxidase-conjugated) was applied thereafter (for 1 h, at room temperature). The membranes were washed and probed using enhanced chemiluminescence (Merck Millipore, Billerica, MA) and autoradiographed using One gel Analysis Apparatus (Furi Science & Technology Co., Shanghai, China). The anti-GAPDH antibody was purchased from Beyotime Biotechnology Corp. (Nanjing, China), the anti-collagen-I antibody was purchased from Novus Biologicals (Centennial, CO), and all other antibodies were purchased from Abcam (Cambridge, UK).

### Statistical analysis

Differences between treatment groups were determined using one-way ANOVA (StatView 5.0.1 software, Abacus Corporation, Baltimore, MD). Tukey’s *post hoc* test was applied when significant differences were noted. Data are shown as the mean ± standard error (SE). All statistical tests were considered significant at *p*< .05.

## Results

### Effects of FFNT25 on physiological and biochemical parameters following UUO

As shown in Table 3, UUO rats had significantly decreased (*p*< .05) body weights and left/right kidney weight (KW) ratios compared to control rats. FFNT25 significantly increased left/right KW ratios following UUO. There was no significant difference (*p*> .05) in renal function indicators including serum Cr, urine Cr, urine Cr/serum Cr ratio, urine NAG, and urine NAG/Cr ratio.

**Table 3.** Physiological and biochemical parameters of rats.

	Control	Sham	UUO	UUO + FFNT25
Initial body weight (g)	207.1 ± 1.5	204.6 ± 1.3	209.4 ± 1.2	209.0 ± 1.6
Final body weight (g)	342.1 ± 2.3	346.8 ± 4.0	308.6 ± 4.4*	297.8 ± 5.0*
Left/right KW	1.036 ± 0.013	1.015 ± 0.029	0.849 ± 0.041*	1.019 ± 0.013^†^
Serum Cr (mg/dL)	1.068 ± 0.042	0.952 ± 0.037	1.216 ± 0.148	1.296 ± 0.072
Urine Cr (mg/dL)	90.613 ± 5.169	81.620 ± 8.496	96.847 ± 9.551	101.278 ± 8.004
Urine Cr/serum Cr	88.410 ± 7.053	87.343 ± 10.202	88.402 ± 13.112	78.952 ± 6.504
Urine NAG (ng/mL)	20.118 ± 2.195	17.502 ± 1.409	20.220 ± 2.598	21.406 ± 2.542
Urine NAG/Cr (ng/mg)	2.283 ± 0.296	2.345 ± 0.335	2.424 ± 0.615	2.238 ± 0.371

KW: kidney weight; Cr: creatinine; NAG: N-acetyl-β-D-glucosaminidase.

Data are presented as mean ± standard error (SE) (*n* = 8).

*p<.05 vs. control.

^†^*p*<.05 vs. UUO group.

### FFNT25 attenuates UUO-induced renal pathology

**Figure 1.**
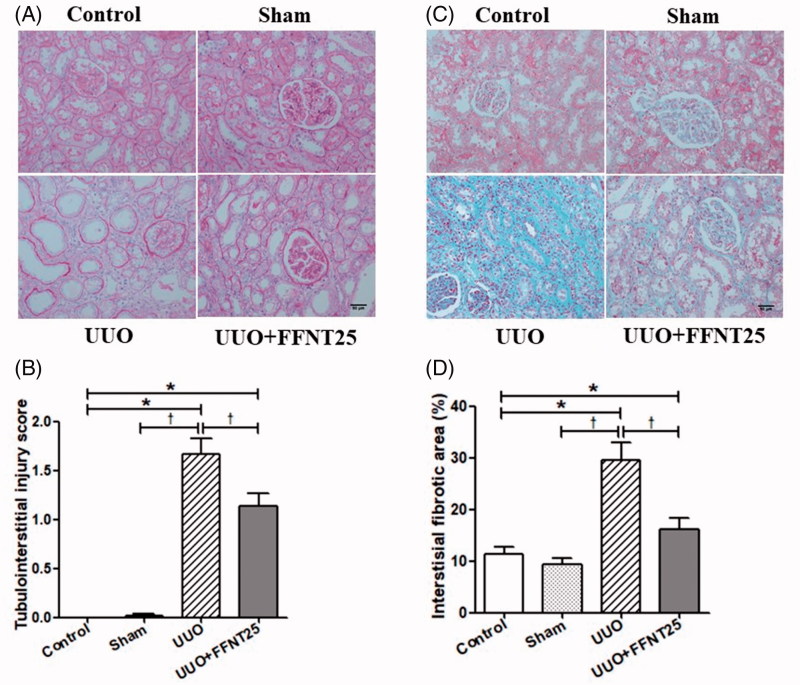
FFNT25 attenuated unilateral ureteral obstruction (UUO)-induced renal pathology in Sprague-Dawley rats. (A) Periodic acid-Schiff (PAS) staining was performed to identify tubulointerstitial injury in kidney sections (×400). (B) Tubulointerstitial injury scores. (C) Masson staining was performed to identify tubulointerstitial fibrosis in kidney sections (×400). (D) Relative percentages of tubulointerstitial fibrosis. Data are presented as means ± SE (*n* = 8). *p<.05 vs. Control; ^†^*p*<.05 vs. UUO group. Scale bar = 50 μm.

PAS and Masson’s trichrome staining were performed to investigate the therapeutic effect of FFNT25 on renal pathology. PAS staining indicated renal pathological alterations, such as interstitial edema, tubular dilatation, epithelial desquamation, and loss of brush border, in UUO rats (Figure 1(A)). UUO rats had higher renal tubulointerstitial injury scores compared to rats in the other groups (Figure 1(B)). Interestingly, FFNT25 significantly improved the pathological alterations and decreased renal tubulointerstitial injury scores in UUO rats. Similarly, as shown by Masson’s staining, FFNT25 dramatically attenuated collagen fibril formation (i.e., ECM deposition) in the kidneys of UUO rats (Figure 1(C,D)).

### FFNT25 decreases UUO-induced ECM deposition

**Figure 2.**
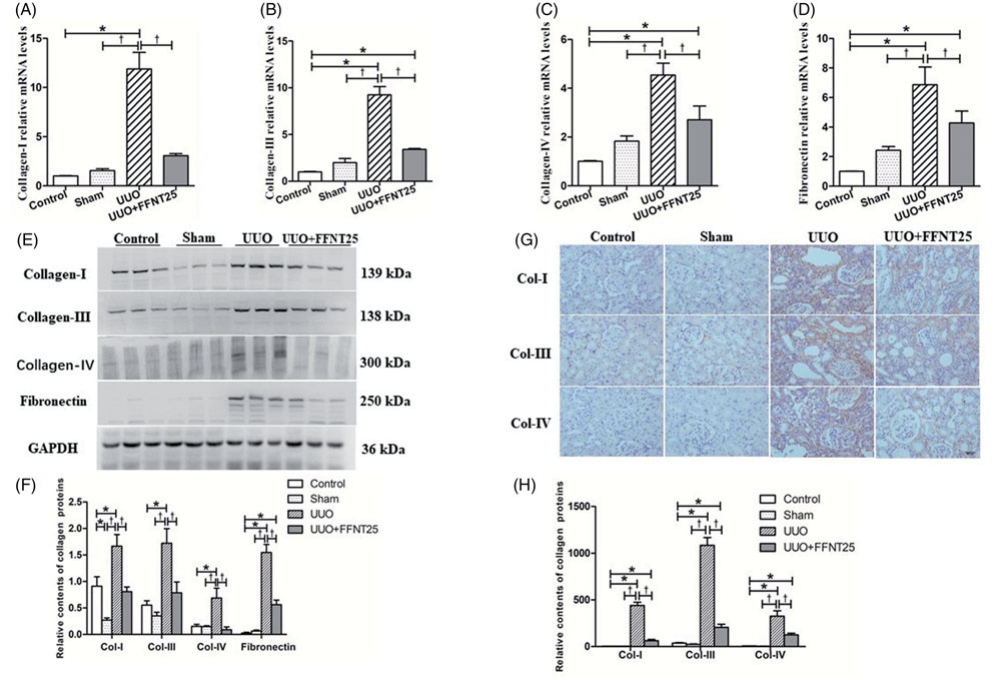
FFNT25 inhibited unilateral ureteral obstruction (UUO)-induced collagen expression in rat kidneys. Real-time qPCR, western blot analysis, and immunohistochemical staining (×400) were conducted to determine renal mRNA and protein expressions of collagen-I (A, E, F, G, H), collagen-III (B, E, F, G, H), collagen-IV (C, E, F, G, H), and fibronectin (D, E, F). Data are presented as means ± SE (*n* = 8). *p<.05 vs. Control; ^†^*p*<.05 vs. UUO group. Scale bar = 50 μm.

ECM is mainly composed of collagen-I, collagen-III, collagen-IV, and fibronectin [30]. We performed real-time qPCR, western blot analysis, and immunohistochemical staining to determine the levels of each of the ECM markers in kidneys following UUO. FFNT25 decreased both mRNA and protein levels of collagen-I, collagen-III, collagen-IV, and fibronectin in the kidneys of UUO rats (Figure 2(A–H)).

### Effects of FFNT25 on UUO-induced myofibroblast activation

**Figure 3.**
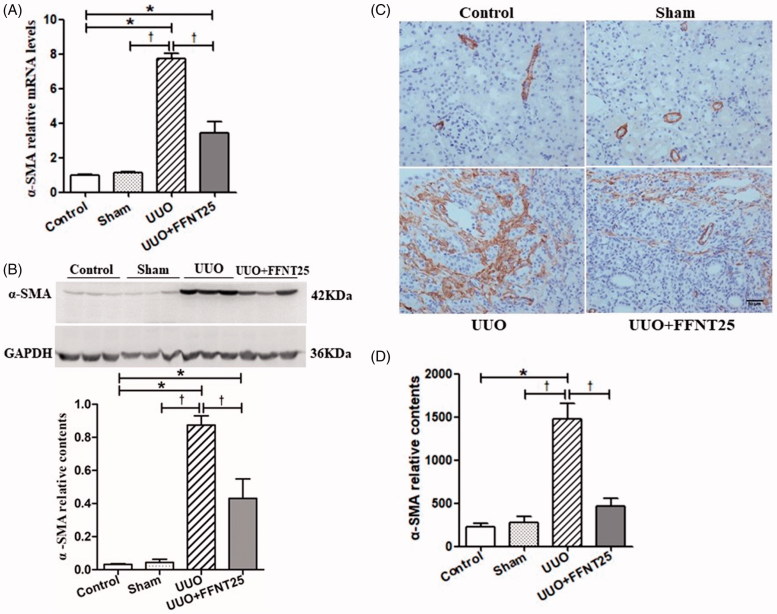
FFNT25 reduced unilateral ureteral obstruction (UUO)-induced activation of myofibroblasts. Renal expression of α-smooth muscle actin (α-SMA) was measured by real-time qPCR (A), western blot analysis (B), and immunohistochemical staining (C and D; ×400). Data are presented as means ± SE (*n* = 8). *p<.05 vs. Control; ^†^*p*<.05 vs. UUO group. Scale bar = 50 μm.

While the origin of myofibroblasts remains controversial [9,10], myofibroblasts have been identified as the dominant collagen-producing cells in renal fibrosis [6]. In this study, UUO rats had significantly increased α-SMA mRNA expression, indicative of increased myofibroblasts in the kidney [11], compared to the rats in the other groups (Figure 3). As expected, FFNT25 significantly decreased kidney α-SMA mRNA expression in the UUO rats. Similar results were observed for western blot analysis and immunohistochemical staining (Figure 3(A–D)).

### FFNT25 reduces UUO-induced PAI-1 expression

**Figure 4.**
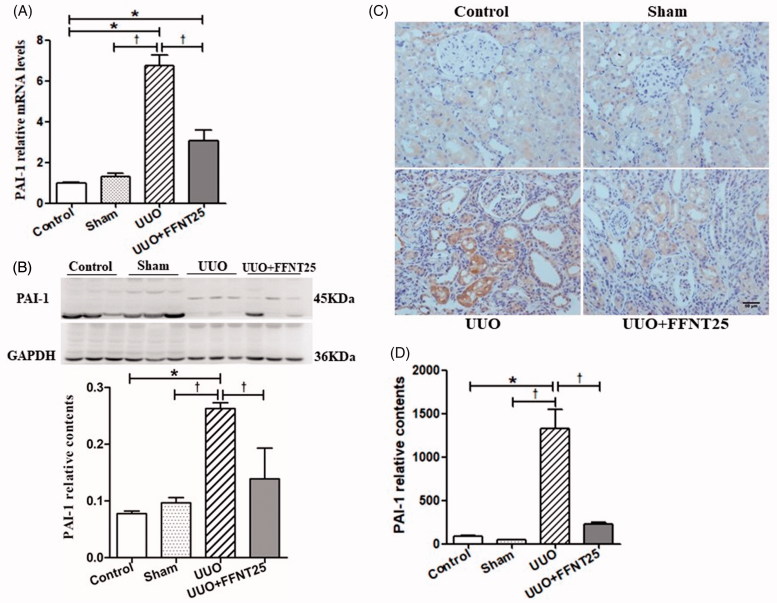
FFNT25 ameliorated unilateral ureteral obstruction (UUO)-induced expression of plasminogen activator inhibitor-1 (PAI-1). Renal expression of PAI-1 was measured by real-time qPCR (A), western blot analysis (B), and immunohistochemical staining (C and D; ×400). Data are presented as means ± SE (*n* = 8). *p<.05 vs. Control; ^†^*p*<.05 vs. UUO group. Scale bar = 50 μm.

UUO rats had increased mRNA and protein levels of PAI-1 in their kidneys compared to the control rats (Figure 4(A,B)). Similarly, immunocytochemical staining showed that PAI-1 expression was significantly increased in the kidneys of UUO rats, but significantly decreased in FFNT25-treated UUO rats (Figure 4(C,D)).

## Discussion

Renal fibrosis is tightly associated with ESRD in CKD patients [31]. While the underlying question of ‘how to deal with renal fibrosis’ has been increasingly investigated in the last few years, a limited number of effective therapeutic strategies have been identified for use in clinical practice. Recently, FFNT25, a molecular compound (see detailed properties in Table 1) developed by Sunshine Lake Pharma Co. (Guangdong, China), was shown to have anti-fibrotic properties similar to its parent drug, PFD, in cultured renal BHK-21 fibroblast cells. In a previous study, both 1.7 mM FFNT25 and 5.8 mM PFD effectively inhibited activation of renal fibroblasts within 48 h. In the current study, the anti-fibrosis function of FFNT25 was assessed *in vivo* in SD rats.

We used UUO to induce renal fibrosis in our study. We found that UUO significantly decreased left/right KW ratio, which was reversed by FFNT25 treatment (Table 3). While renal function indicators, such as serum Cr, urine Cr, and urine NAG, were not affected by UUO, we concluded that these indices also estimated the function of the contralateral non-obstructed kidney in the UUO model, which suggests that there is renal compensation. We observed no obvious side effects (e.g., diarrhea) of FFNT25. These results indicate that FFNT25 could be used to alleviate interstitial edema in renal fibrosis.

Tubulointerstitial injury is common in UUO-induced renal fibrosis. Similar to previous reports [27,28], severe tubulointerstitial injuries (such as interstitial edema, renal tubular dilation, brush border loss, and tubular epithelial cell necrosis/loss) were observed in UUO rats in the current study. FFNT25 treatment significantly ameliorated these UUO-induced tubular injuries (Figure 1(A,B)). Therefore, FFNT25 might be used to prevent tubulointerstitial injuries and promote recovery following renal fibrosis.

ECM deposition is a major complication of renal fibrosis. Continuous activation of interstitial myofibroblasts, defects in matrix proteolysis, and abnormal MMPs could induce ECM deposition in the kidney. As a result, ECM replaces the normal organizational structure and suppresses the normal function of the kidney [32,33]. Collagen-I, collagen-III, collagen-IV, and fibronectin have been identified as the major ECM components [6,30,34]. In this study, we measured the amount of renal ECM using Masson’s trichrome staining. As shown in Figure 1(C,D), FFNT25 inhibited ECM deposition in UUO rats. As expected, the mRNA and protein levels of each of these fibrillar proteins was increased in UUO rats compared to control rats, but was decreased in FFNT25 treated UUO rats compared to vehicle treated UUO rats (Figure 2(A–H)).

Physiological levels of ECM are essential for maintaining structural integrity in the kidney [10]. However, major complications are associated with unbalanced ECM production and degradation. In severe and/or persistent renal injury, aberrant fibroblasts differentiate into myofibroblasts, which implies increased ECM production in the context of renal fibrosis [7,35]. While a number of markers have been identified in the literature [36–38], to our understanding, α-SMA is the most sensitive parameter for evaluating myofibroblast differentiation and activation [11,39]. In the present study, α-SMA expression was highly increased in UUO rats compared to control rats, which was significantly decreased by FFNT25. Based on these results, it is reasonable to conclude that FFNT25 restrains myofibroblast activation and accordingly decreases ECM production.

**Figure 5.**
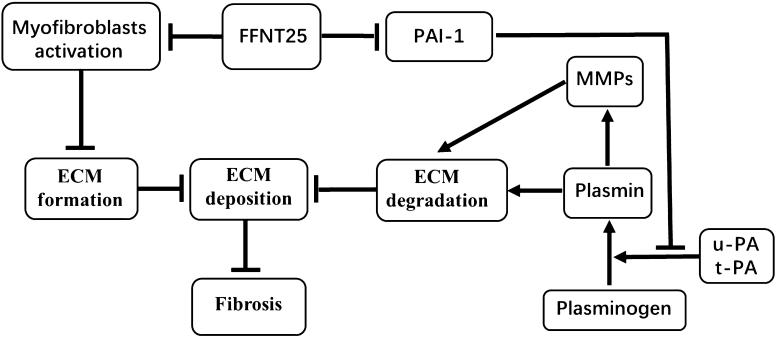
The proposed role of FFNT25 in ameliorating renal fibrosis. FFNT25 attenuates renal fibrosis by: (1) inhibiting myofibroblast activation and preventing the extracellular matrix (ECM) formation and (2) suppressing expression of plasminogen activator inhibitor-1 (PAI-1) and stimulating ECM degradation.

ECM degradation is triggered by two *in vivo* mechanisms: (1) plasminogen activation and (2) MMPs [40,41]. Plasmin can degrade fibrils (e.g., fibronectin and collagen-IV) and activate latent MMPs [42]. PAI-1, the main inhibitor of plasminogen activation, can interact with tissue type plasminogen activator (t-PA) and urokinase-type plasminogen activator (u-PA), thereby decreasing plasmin production (from plasminogen) and consequently inhibiting fibrin degradation (Figure 5). In this study, renal expression of PAI-1 was significantly increased in UUO rats compared to control rats, but was clearly decreased by FFNT25 treatment (Figure 4(A–D)), suggesting that FFNT25 inhibits renal PAI-1. Thus, our findings suggest that FFNT25 has anti-fibrosis properties, including the ability to enhance collagen degradation.

To our knowledge, we are the first to report the use of FFNT25 for ameliorating renal fibrosis *in vivo* using the UUO rat model. FFNT25 inhibited ECM production and promoted ECM clearance as part of its anti-fibrosis effects. Importantly, we did not observe any side effects in FFNT25 treated rats. These results clearly suggest the clinical potential of FFNT25 for managing renal fibrosis. Future studies will investigate the mechanism by which FFNT25 regulates α-SMA and/or PAI-1 in renal fibrosis.
